# Engineering Properties of Green and Ecofriendly Grouting Materials with Different Sand Filling Ratios

**DOI:** 10.3390/ma16020837

**Published:** 2023-01-15

**Authors:** Chuen-Ul Juang, Wen-Ten Kuo

**Affiliations:** Department of Civil Engineering, National Kaohsiung University of Science and Technology, Kaohsiung 807, Taiwan

**Keywords:** green and ecofriendly grouting material, superplasticizers, engineering properties, sand filling ratio

## Abstract

With the active development of offshore wind power worldwide, the development of a green and ecofriendly grouting material (GEGM) has garnered global attention. Such a material must also be developed in Taiwan. Therefore, in this study, a series of environmentally friendly recycled materials were mixed in different proportions to develop a GEGM which can be implemented in the future construction of offshore wind turbines. To evaluate the mechanical properties of the GEGM, low water-to-binder (W/B) ratios (i.e., 0.21, 0.27, and 0.35) were used; cement was replaced with fixed amounts (20%) of ground granulated blast-furnace slag and fly ash; 2%, 2.5%, and 3% superplasticizers (SPs) were added; and two levels of sand content (60% and 70%) were used. The setting time of the GEGM was used to evaluate its workability; its compressive strength and flexural strength were used to evaluate its mechanical properties; and its sulfate resistance, length changes, and four-terminal resistance were used to evaluate its durability. The relationship between the durability and drying shrinkage of the GEGM was subsequently evaluated, and the ratio of final to initial setting times (F/I value) was calculated to determine the effects of the amount of SP added on workability. The highest F/I value (7.7) was achieved when 2 wt.% modified lignin sulfonate (MLS) was added because of the high viscosity of MLS, which compromised the workability of the concrete. The optimal compressive strength (83.62 MPa) was achieved when a W/B ratio of 0.21 was used, when the sand content was 70%, and when a 2% polycarboxylate superplasticizer (PCE) was added, whereas the optimal flexural strength (20.86 MPa) was achieved when 2.5% PCE was added. According to the nondestructive test results and the R2 value (>0.7) obtained from regression analyses of mechanical properties, the study results are reliable and may serve as a reference for future construction projects.

## 1. Introduction

For years, Taiwan has relied on imported petroleum to generate electricity. However, as the costs of raw materials have increased worldwide, regions that have long relied on imported raw materials to generate power have suffered detrimental effects [1]. Therefore, faced with such a change in the global environment, Taiwan must actively promote energy transition and produce renewable energy on its own [2]. According to a global assessment of offshore wind farms, Taiwan has been identified as one of the most suitable countries for the development of offshore wind energy [3,4,5,6,7]. The prevalence of high-performance concrete (HPC) has improved the construction outcomes of numerous civil engineering projects, attracting comprehensive research in this field [8,9]. Ground granulated blast-furnace slag (GGBFS), which undergoes pozzolanic reactions [10,11,12,13,14,15], is an effective substitute for cement materials to increase the porosity and durability of concrete [16,17]. Zaid et al. [18] fabricated HPC from recycled concrete aggregates and GGBFS, in which cement was replaced with 25% GGBFS. The HPC produced after being cured for 28 and 56 days showed a reduced compressive strength and a considerably reduced water absorption rate (13%). Such a reduction in water absorption rate was attributable to the use of GGBFS, which displayed an increased degree of fineness, and the hydration products produced via GGBFS hydration filled the cracks of the regenerated concrete aggregates [19]. Biskri et al. [20] noted that the optimal cement replacement rate of GGBFS was 20%. In another study, Nduka et al. [21] used rice husk ash and a low water-to-binder (W/B) ratio of 0.3 to produce HPC. The results indicated that a 10% substitution using rice husk ash was optimal. Bachtiar et al. [22] selected seawater as the mixing water for HPC and discovered that adding seawater and curing concrete in a seawater environment produced concrete with comparatively lower porosity. Mokhtar et al. [23] used nanoscale clay, silicon dioxide, and mixed aggregates to evaluate the performance of HPC after being cured for 28 days. The results indicated that the additives enhanced the mechanical properties of HPC. Given the public’s increased environmental awareness, an increasing number of recycled materials have been used to develop HPC with favorable outcomes [24,25,26,27,28]. In other studies, self-filling concrete, HPC, and reactive powder concrete have been developed on the basis of packing theory. The objective was to achieve optimal particle filling in order to improve the engineering properties and durability [29,30,31,32]. Using packing density theory, Zhang et al. [33] investigated an aeolianite HPC proportion design. They discovered that a water-to-cement ratio of 0.15, a superplasticizer (SP) additive of 1%, and 25.86% substitution with fly ash (FA) produced fluidity of 220 mm, a 28-day flexural strength of 20.26 MPa, and a 28-day compressive strength of 97.09 MPa, thus meeting the property requirements for HPC. Rudnicki [34] used a large amount of furnace-stone powder to replace traditional cement to make high-performance concrete, which was shown to effectively improve the compactness of concrete and can be applied as concrete pavements on roads in the future.

A grouting sleeve is a structure inserted into an assembled structure, and grout is then poured to bind the parts together. Currently, grouting is the most cost-effective method of construction [35,36,37] and is used in offshore wind turbines. According to many researchers, the manner in which grout sleeves are connected is a crucial factor in determining structural mechanics [38,39,40,41]. However, no studies have yet been carried out to investigate the use of grouting materials in offshore wind turbines. As Taiwan is currently seeking to actively develop its offshore wind power, research and development regarding green grouting materials for offshore wind turbines must urgently be carried out. The outcomes may serve as a reference for other Southeast Asian countries that are seeking to develop offshore wind turbines.

## 2. Materials and Methods

### 2.1. Green and Ecofriendly Grouting Material Preparation

This section lists the materials used in this study and their providers. Type I Portland cement (which met the ASTM C150 standard [42]) was provided by Southeast Cement Corporation (Kaohsiung, Taiwan). Recycled, environmentally friendly class F coal FA (which met the ASTM C618 standard [43]) was provided by the Taiwan Power Company (Kaohsiung, Taiwan). GGBFS (which met the ASTM C989 standard [44]) was provided by China Steel Corporation (Kaohsiung, Taiwan). Table 1 lists the basic compositions of these cementitious materials, which were used in this study to produce a green and ecofriendly grouting material (GEGM). All the aggregates (coarse and fine) met the ASTM C33 [45] gradation curve requirements. In addition, all the SPs, namely, polycarboxylate superplasticizer (PCE), sulfonated naphthalene formaldehyde (SNF) condensates, and modified lignin sulfonate (MLS) salts, were provided by Yo Rich (Taipei, Taiwan). PCE is a light-yellow liquid with a water reduction rate of 26.9%; SNF is a dark-brown liquid with a water reduction rate of 23.6%; and MLS is a brown liquid with a pungent odor and a water reduction rate of 12.2%.

### 2.2. Mixing Proportions

To prepare the GEGM, W/B ratios of 0.21, 0.27, and 0.35 were used, and 2%, 2.5%, and 3% SP (PCE, SNF, and MLS) were added, respectively, to explore the effects of different SPs on the GEGM. Two levels of sand content (i.e., 70% and 60%) were used as experimental variables to investigate the specimen density, and GGBFS and FA were used to replace cement at a fixed amount (20%) to reduce the environmental impact of the prepared material. Table 2 lists the test ratios.

### 2.3. Methods

The GEGM was prepared by mixing different materials according to the procedure specified in ASTM C305 [46]. Specifically, 20% of the cement was replaced with GGBFS and FA, and W/B ratios of 0.21, 0.27, and 0.35 were used. The setting time (ASTM C807) [47] was used to explore the workability of the prepared GEGM, and the mechanical properties were determined. We put the samples into saturated lime solution for 1 day, 7 days, 28 days, and 56 days, and analyzed the compressive strength (ASTM C109) [48], flexural strength (ASTM C348) [49], ultrasonic wave speed (ASTM C597) [50], and water absorption rate (ASTM C1585) [51] of the specimens measuring 5 × 5 × 5 cm^3^. Numerical analyses were performed to establish the correlations between the aforementioned parameters. Durability was assessed using sulfate resistance (ASTM C1012) [52], length changes (ASTM C596) [53], and four-terminal resistance (ASTM C876) [54].

## 3. Results and Discussion

### 3.1. Workability of the GEGM

**Figure 1:**
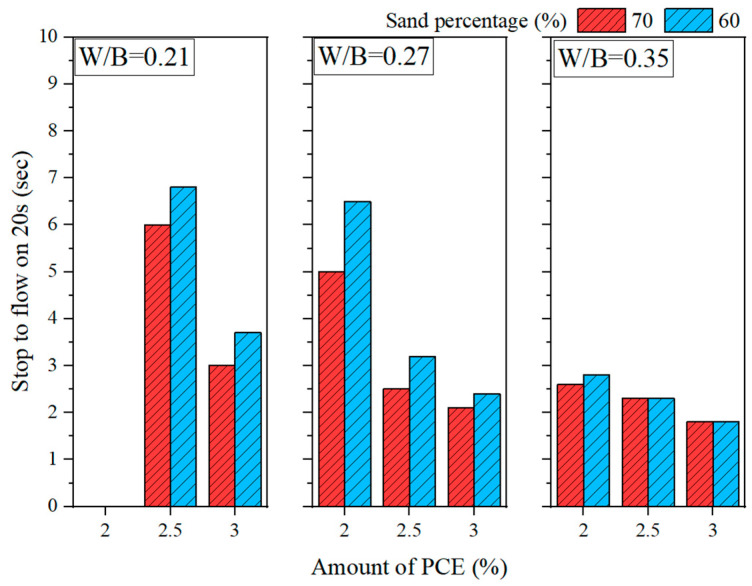
The time required for the flow of PCE to 20 cm.

**Figure 2:**
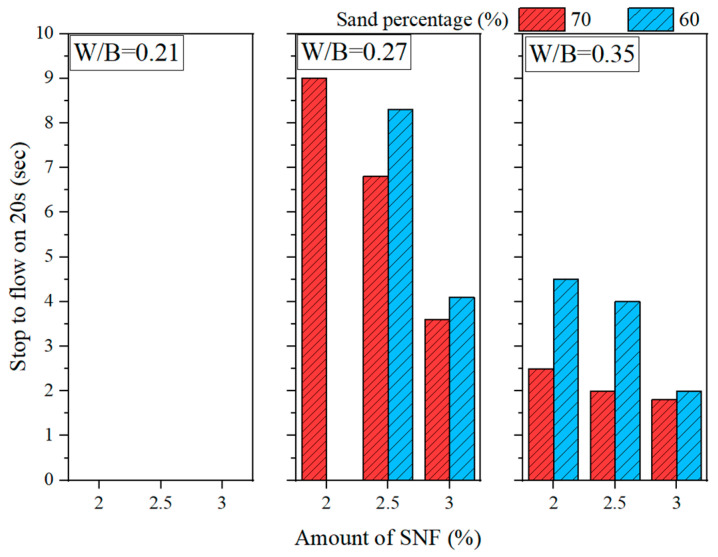
The time required for the flow of SNF to 20 cm.

**Figure 3:**
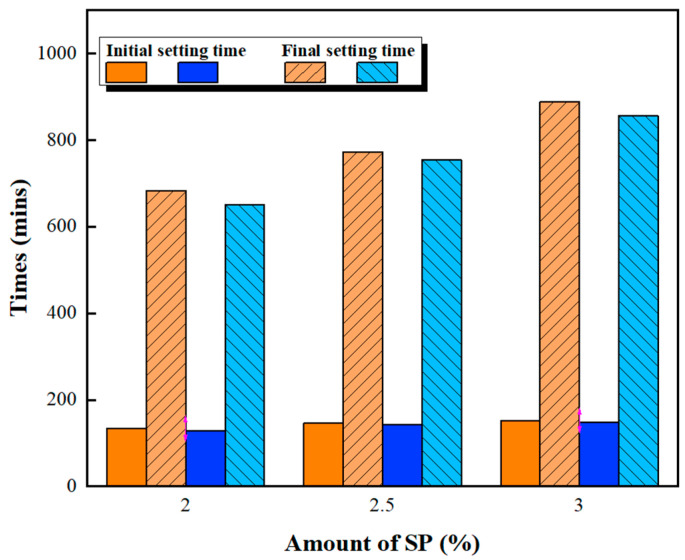
Setting time of green and ecofriendly grouting material with W/B = 0.21.

**Figure 4:**
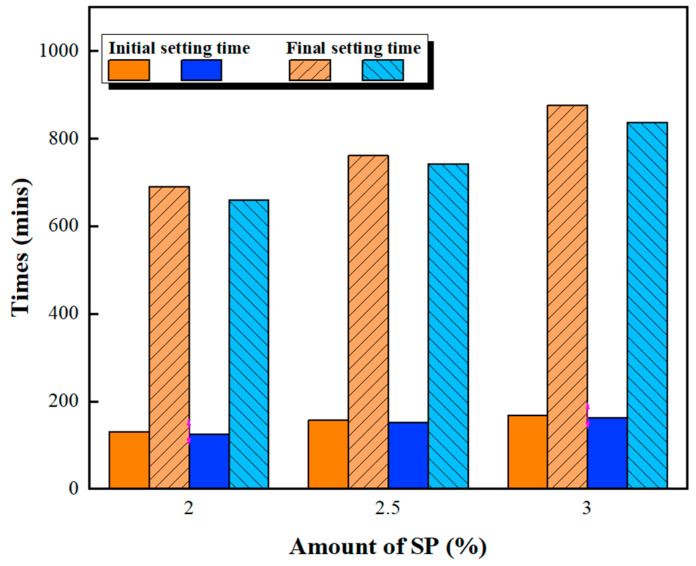
Setting time of green and ecofriendly grouting material with W/B = 0.27.

**Figure 5:**
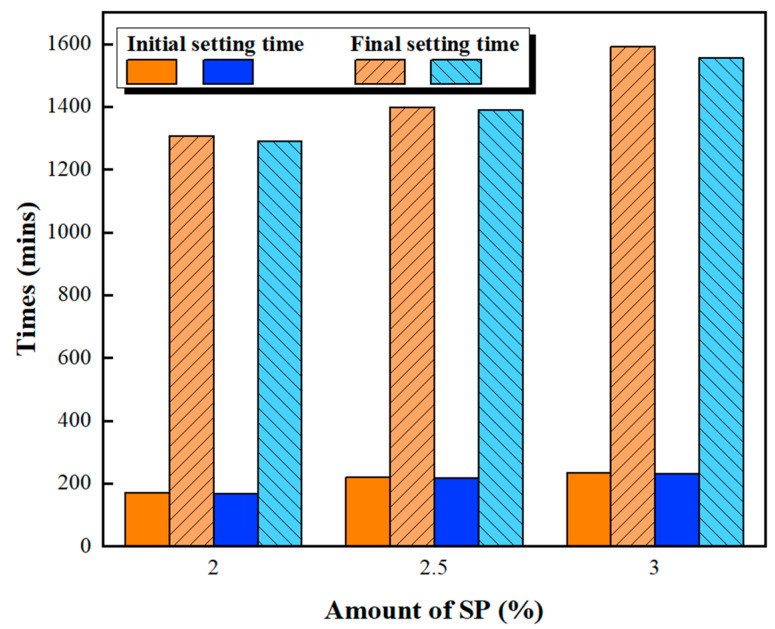
Setting time of green and ecofriendly grouting material with W/B = 0.35.

**Figure 6:**
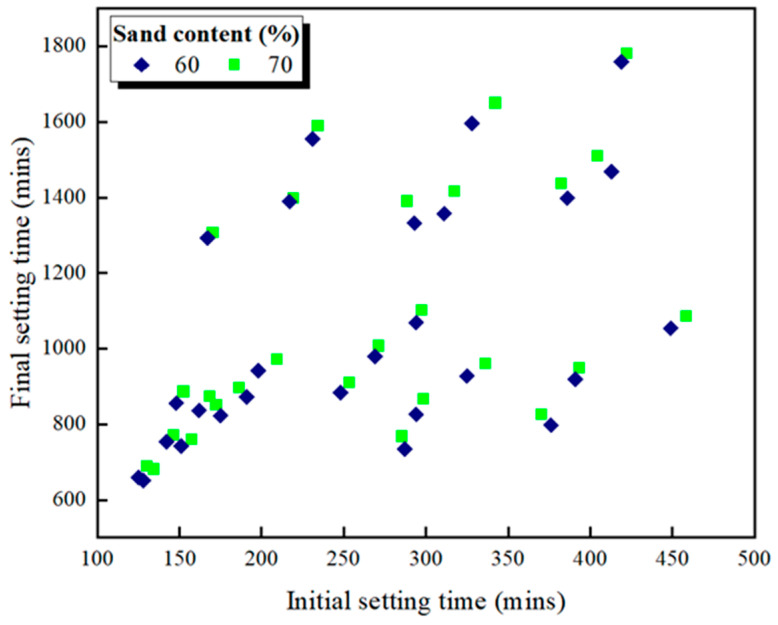
Setting time of green and ecofriendly grouting material.

Figure 1 and Figure 2 show the slump fluidity experiments for PCE and SNF; because the slurry of MLS itself was less fluid, the experiment could not be carried out. The experimental results show that, in the case of low W/B, the addition of 2% could not produce fluidity; therefore, these data are not displayed. With W/B = 0.35, the sand content had little effect on fluidity, and the best fluidity was achieved with W/B = 0.27 and the addition of 3%. Figure 3, Figure 4 and Figure 5 depict the results regarding the setting time. Both levels of sand content (i.e., 60% and 70%) exhibited consistent setting-time trends (i.e., the setting time increased with the W/B ratio). The longest setting time was recorded with 3 wt.% SNF when the sand content was 70%; the initial setting time was 458 min, and the final setting time was 1793 min. These results agree with those of Niu et al. [55], who reported that sulfonated naphthalene SPs increased the setting time of cement materials. When the initial and final setting times were similar, the development of concrete strength was affected. For instance, an increase in the W/B ratio from 0.27 to 0.35 increased the initial setting time by 23.4% to 48.5% and the final setting time by 4.9% to 13.6%. Similarly, an increase in the W/B ratio from 0.21 to 0.35 increased the initial setting time by 81.3% to 184.6% and the final setting time by 5.7% to 35.8%. Therefore, both the initial and final setting times were obtained. To determine the effects of the amount of SP added on workability, the ratio of the final to initial setting times (F/I) was calculated. The highest F/I value was 7.7, which was achieved when 2 wt.% MLS was added, because of the high viscosity of MLS [56]. Because SPs were adsorbed on the surfaces of the cement particles and because an increase in the amount of SP added caused an increase in their absorption, the formation of chains between hydration products was delayed, resulting in retarded setting and poor workability. Hence, the flow comparison results were obtained. Figure 6 depicts the setting times achieved when sand content levels of 60% and 70% were used. Regardless of the various effects of SPs on the setting time, the sand content exhibited a minimal effect on the setting time of the GEGM, suggesting that the sand content only affected the flow of the GEGM and not the setting time. This was due to the high level of fine aggregate content, which retained water and thus resulted in retarded setting during the final setting process.

### 3.2. Mechanical Properties of the GEGM

#### 3.2.1. Compressive Strength of the GEGM

**Figure 7:**
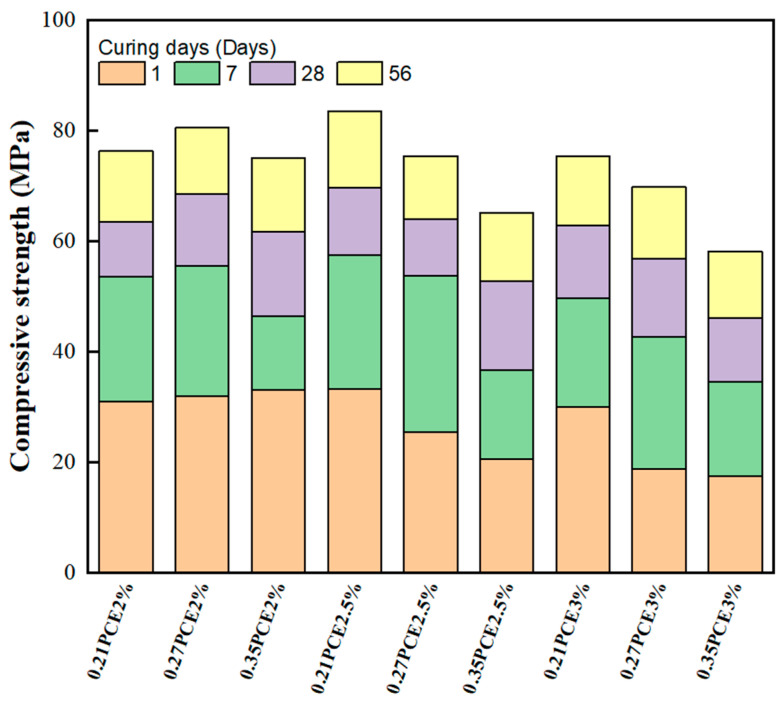
Compressive strength of green and ecofriendly grouting material with PCE.

**Figure 8:**
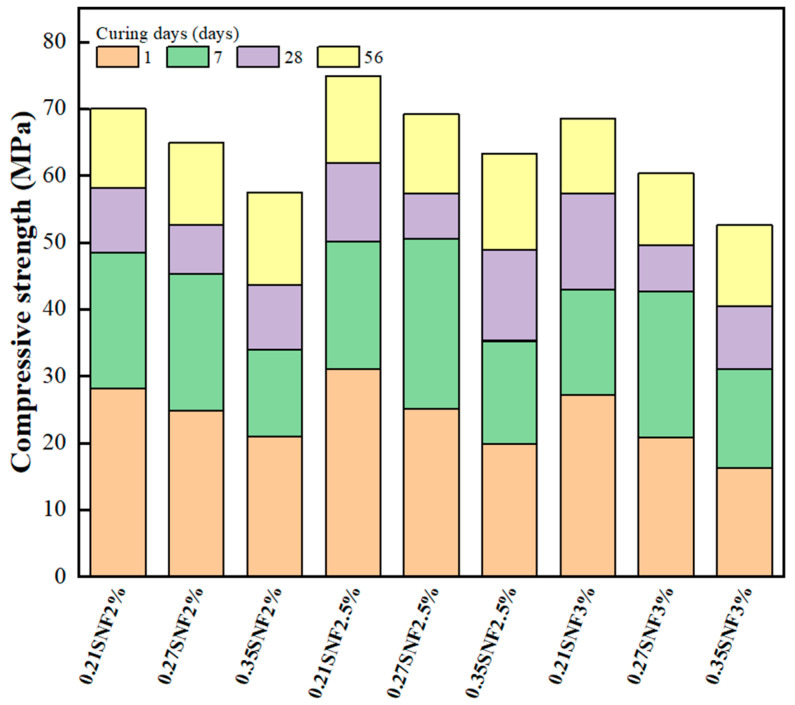
Compressive strength of green and ecofriendly grouting material with SNF.

**Figure 9:**
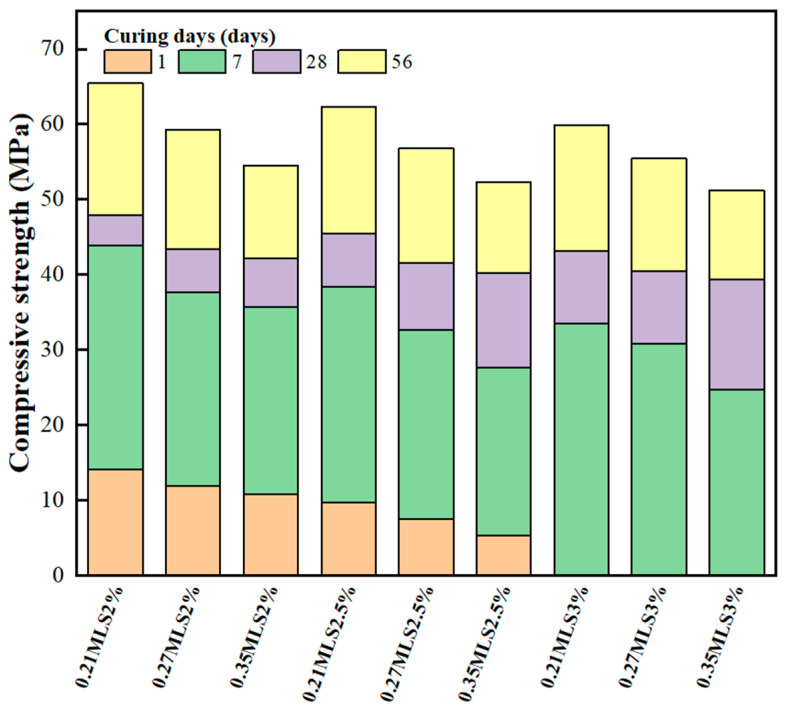
Compressive strength of green and ecofriendly grouting material with MLS.

**Figure 10:**
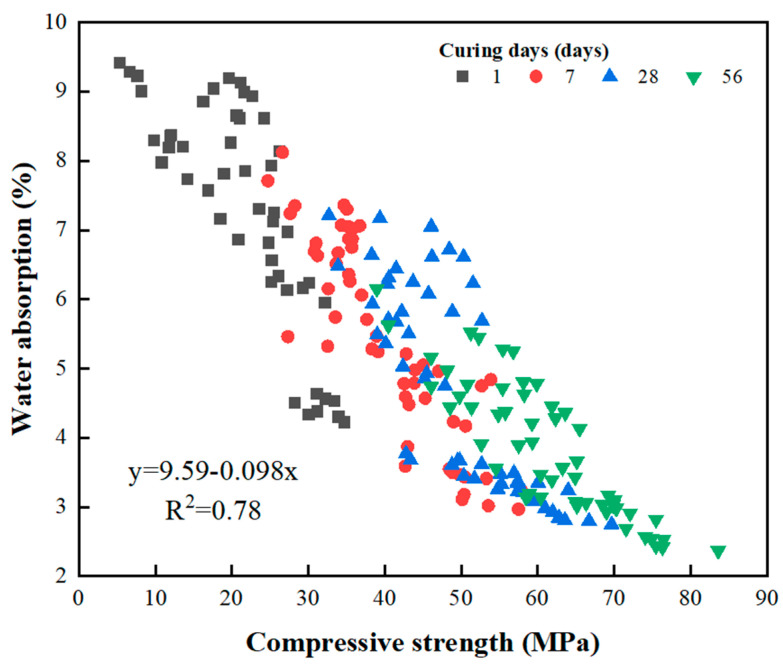
Linear regression analysis with compressive strength and water absorption of green and ecofriendly grouting material.

**Figure 11:**
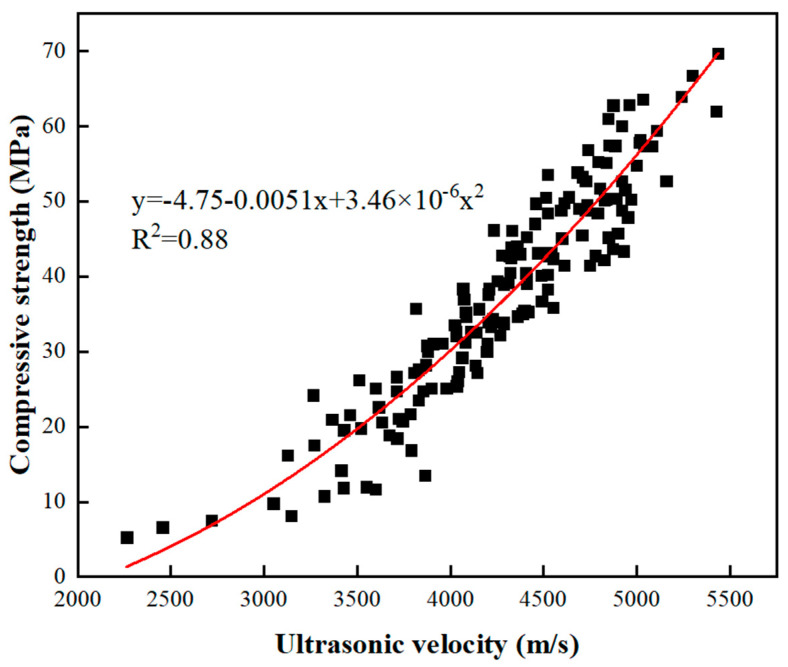
Regression analysis of compressive strength and ultrasonic wave velocity of green and ecofriendly grouting material.

Compressive strength is one of the most crucial properties of the GEGM. Figure 7, Figure 8 and Figure 9 depict the results of compressive strength experiments conducted with the three aforementioned SPs. The optimal compressive strength (83.62 MPa) was achieved when the W/B ratio was 0.21, when the sand content was 70%, and when 2% PCE was added. When the W/B ratio increased, the compressive strength decreased. For instance, for PCE, when the W/B ratio increased from 0.27 to 0.35, the compressive strength decreased by approximately 11.7% to 16.6%. For SNF, when the W/B ratio increased from 0.27 to 0.35, its compressive strength decreased by approximately 8.6% to 12.8%. Finally, for MLS, when the W/B ratio increased from 0.21 to 0.27 and from 0.21 to 0.35, its compressive strength decreased by approximately 7.5% to 9%. Hence, the three SPs yielded consistent results, because the addition of the SP agents enhanced the workability of the GEGM and made the slurry sticky. However, the addition of these agents reduced the compressive strength [57,58,59]. The main reason for this is that the addition of too much of these agents causes molecules to adsorb on the surface of the particles, which will hinder the surface tension of the cement mortar and cause many small air bubbles to form, resulting in a decrease in the compressive strength. For PCE and SNF, when curing was performed for 1–7 days, the compressive strength increased considerably. However, when curing was performed for more than 28 days, the compressive strength decreased. For MLS, a different outcome was observed; that is, when the MLS content was increased to 3%, the setting was retarded, which made the compressive strength unobservable on the first day of curing. Because all three SP agents used in this study exhibited consistent developmental trends, a regression analysis of their compressive strengths and water absorption rates was performed. As shown in Figure 10, a linear regression equation was used. The results revealed that *y* = 9.59 − 0.098*x* and *R*^2^ = 0.78, indicating the reliability of the results. According to Figure 10, when the compressive strength increased, the water absorption rate decreased. As shown in Figure 11, to identify the regression relationship between the ultrasonic wave speed and compressive strength, the ultrasonic wave speed was measured using a nondestructive testing method. Additionally, the prediction of compressive strength through ultrasonic wave speeds revealed an *R*^2^ of 0.88, indicating high reliability. When curing was performed for 28 to 56 days, the sand content of 70% yielded the highest compressive strength. At said content, the longer the curing duration was, the greater the difference in compressive strength was. This is because concrete with 70% sand content had low porosity, resulting in a more compact specimen and higher compressive strength.

#### 3.2.2. Flexural Strength of the GEGM

**Figure 12:**
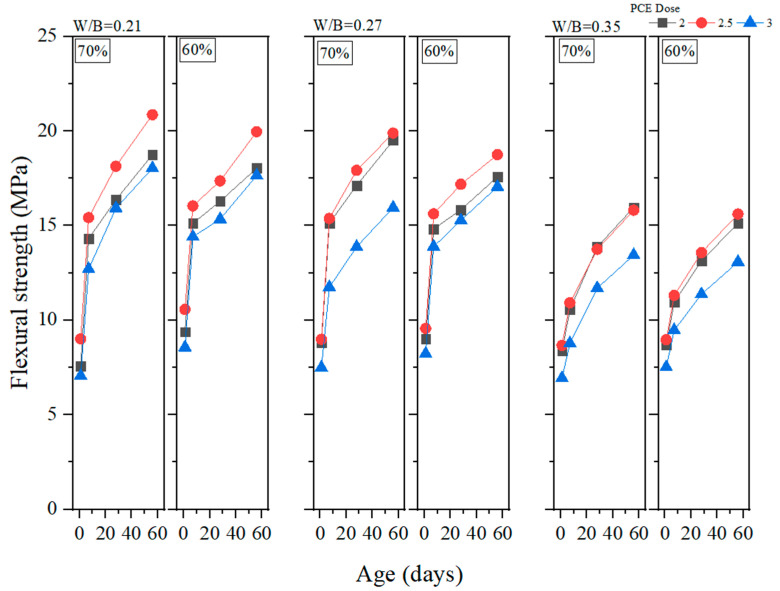
Flexural strength of green and ecofriendly grouting material with PCE.

**Figure 13:**
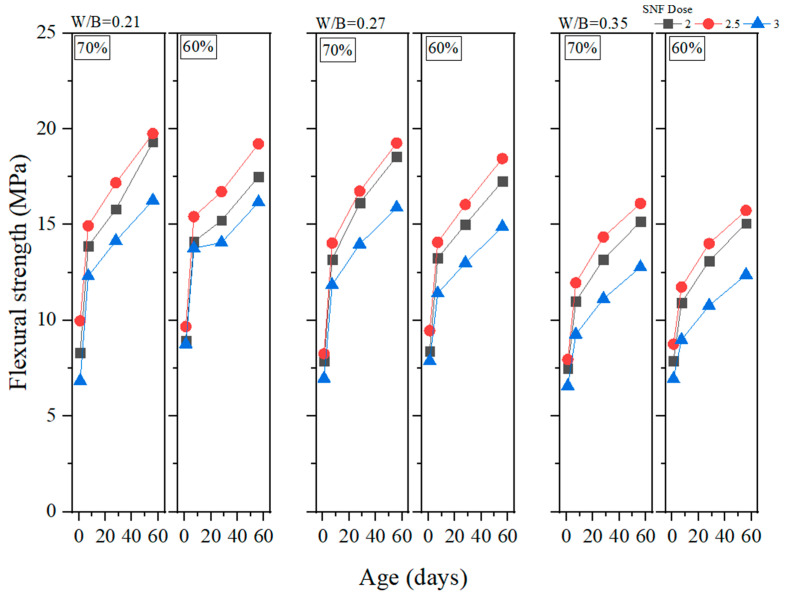
Flexural strength of green and ecofriendly grouting material with SNF.

**Figure 14:**
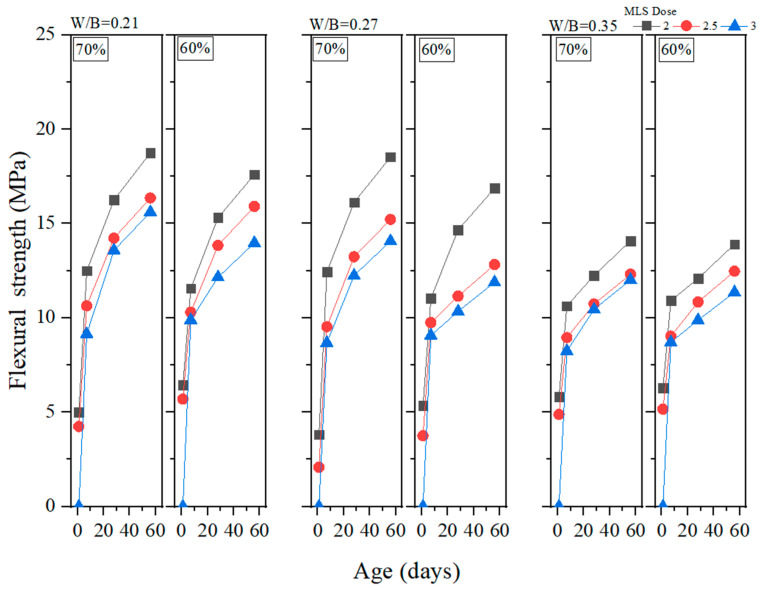
Flexural strength of green and ecofriendly grouting material with MLS.

**Figure 15:**
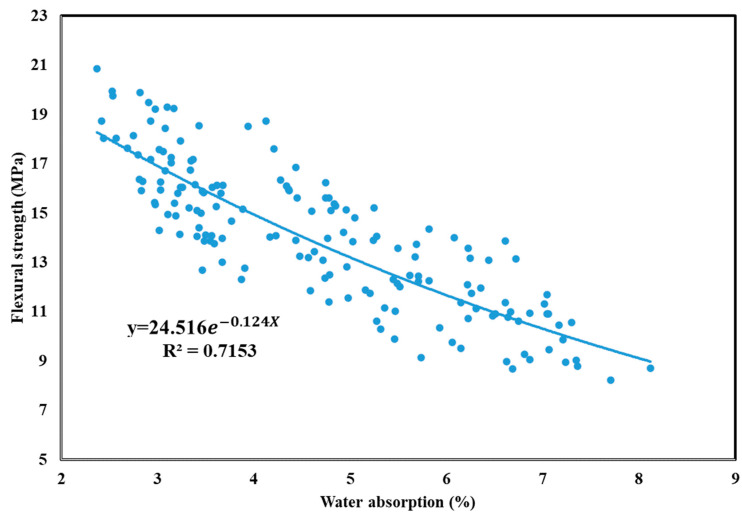
Regression analysis of flexural strength and water absorption of green and ecofriendly grouting material.

Flexural strength is a crucial property of GEGMs which should be examined if they are to be used in offshore wind turbines. This is because the materials used in a wind turbine must be able to effectively withstand the effects of external forces at sea. In this study, flexural strength experiments were conducted on PCE, SNF, and ML, and the results are presented in Figure 12, Figure 13 and Figure 14, respectively. As shown in Figure 10, the addition of 2.5% PCE yielded optimal results, especially at low W/B ratios. When curing was performed for 56 days, the flexural strength reached 20.86 MPa, the highest value reached among all experiments. As shown in Figure 13, the addition of 2.5% SNL yielded optimal results, with an increase in the W/B ratio decreasing the flexural strength. As shown in Figure 14, the addition of 2% ML yielded optimal results. The figure also depicts the effects of the sand content and how these effects became more pronounced at low W/B ratios. When the W/B ratio increased to 0.35%, the sand content had a minor effect on flexural strength. When curing was performed for 1–7 days, 60% sand content produced a compressive strength that was 4% to 24.1% higher than that produced with 70% sand content. When curing was performed for 28–56 days, 60% sand content produced a compressive strength that was 0.5% to 4.3% lower than that produced with 70% sand content. Therefore, in this study, regression analyses of flexural strengths and water absorption rates were performed, and the results are depicted in Figure 15. Here, a correlation (*R*^2^) of 0.71 was observed. Although this value is less than 0.8, it still reached a favorable level of reliability.

### 3.3. Durability of the GEGM

Durability is a critical factor in civil engineering. To ensure the safety of buildings and to extend their service life, the durability of the materials used to build them must be scrutinized. In this study, durability tests were conducted to investigate the sulfate resistance, length changes, and four-terminal resistance of the GEGM specimens.

#### 3.3.1. Sulfate Resistance of the GEGM

**Figure 16:**
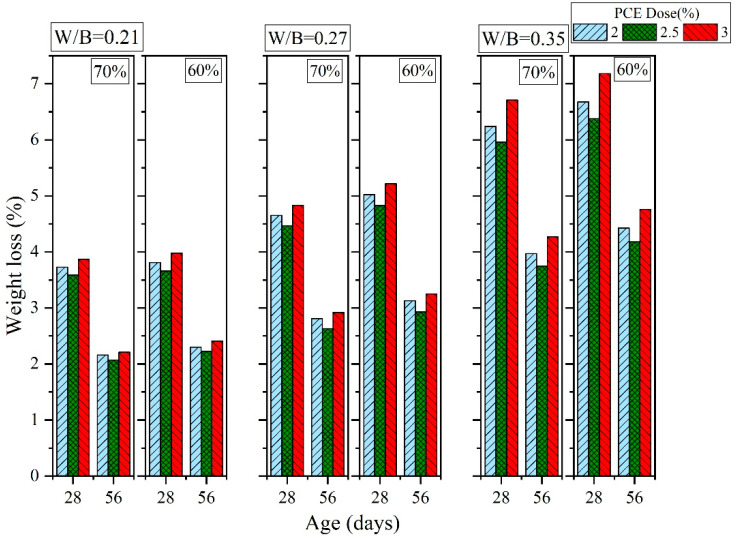
Sulfate resistance of green and ecofriendly grouting material with PCE.

**Figure 17:**
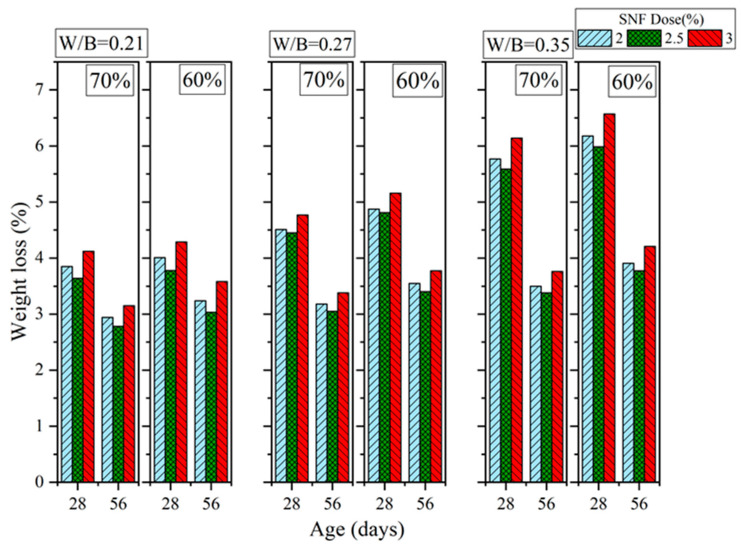
Sulfate resistance of green and ecofriendly grouting material with SNF.

**Figure 18:**
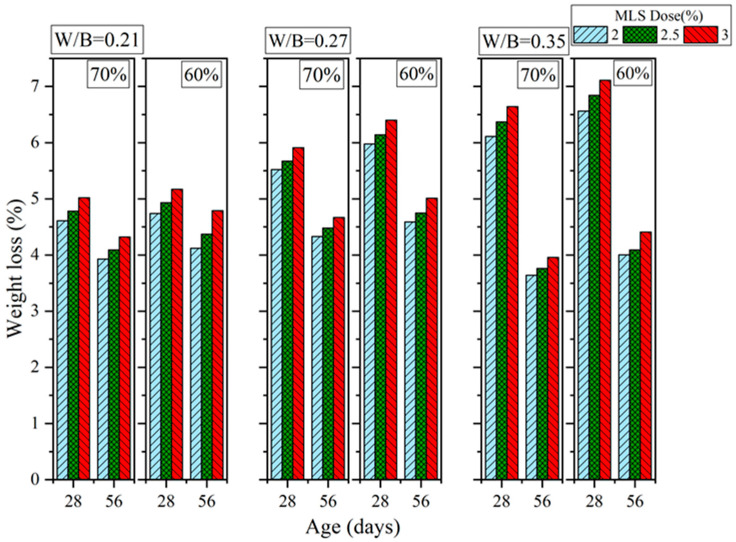
Sulfate resistance of green and ecofriendly grouting material with MLS.

Figure 16, Figure 17 and Figure 18 show the results of the sulfate resistance experiments. Specimens with 70% sand content exhibited greater sulfate resistance than those with 60% sand content. This is because the specimens with 70% sand content were denser and thus more resistant to sulfates; these specimens also exhibited decreased weight loss rates. For PCE, the weight loss rate was 3.59% to 3.87% when the W/B ratio was 0.21, when 2% to 3% PCE was added, when curing was performed for 28 days, and when the sand content was 70%. However, when the sand content was 60% (while keeping the other variables constant), the weight loss rate reached 3.66% to 3.98% (an increase of 1.95% to 2.84%). When the curing duration was increased to 56 days, the 70% and 60% sand contents resulted in weight loss rates of 2.07% to 2.21% and 2.22% to 2.41%, respectively (the latter was 6.48% to 9.04% higher than the former). Moreover, the addition of 2.5% PCE and SNF resulted in the lowest weight loss rates. These results are consistent with those obtained regarding mechanical properties because the additives made the GEGM specimens denser and thus more resistant to sulfates. In terms of MLS, the optimal sulfate resistance was achieved when 2% MLS was added. Because MLS induced retarded setting in the GEGM, and therefore retarded hydration, only a small amount of MLS should be added to avoid compromising the engineering properties and effectiveness of the GEGM.

#### 3.3.2. Length Changes of the GEGM

**Figure 19:**
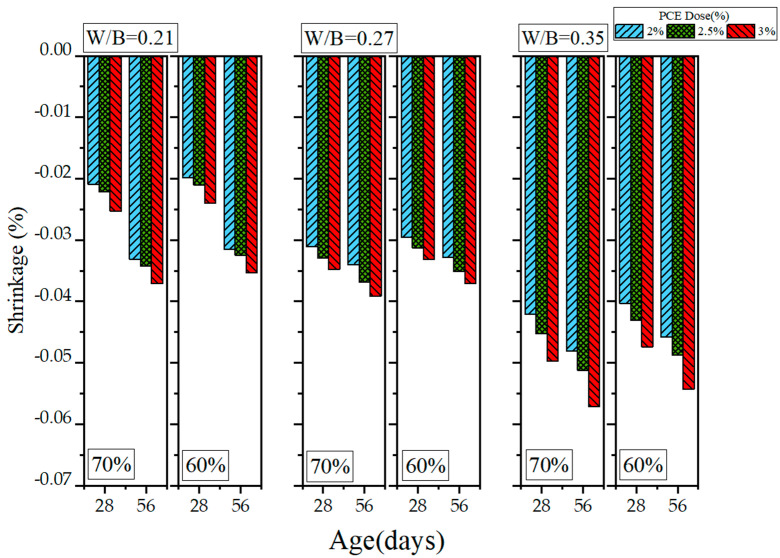
Length changes of green and ecofriendly grouting material with PCE.

**Figure 20:**
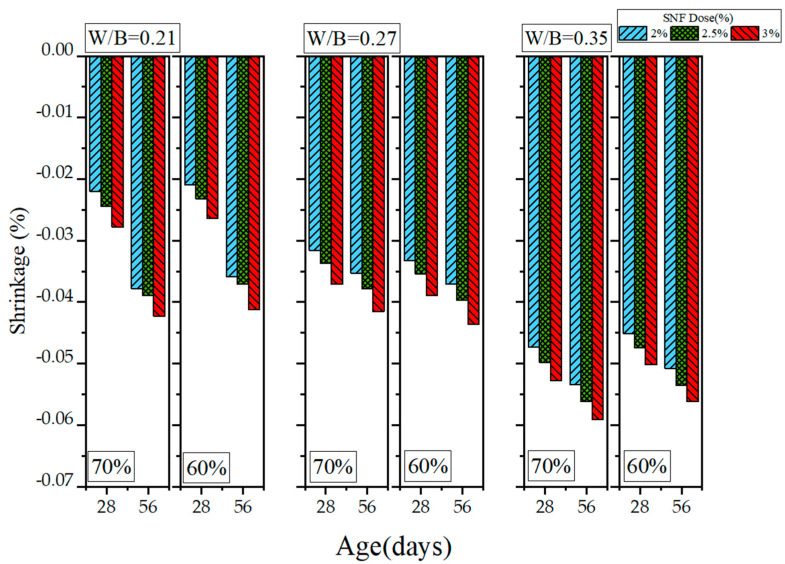
Length changes of green and ecofriendly grouting material with SNF.

**Figure 21:**
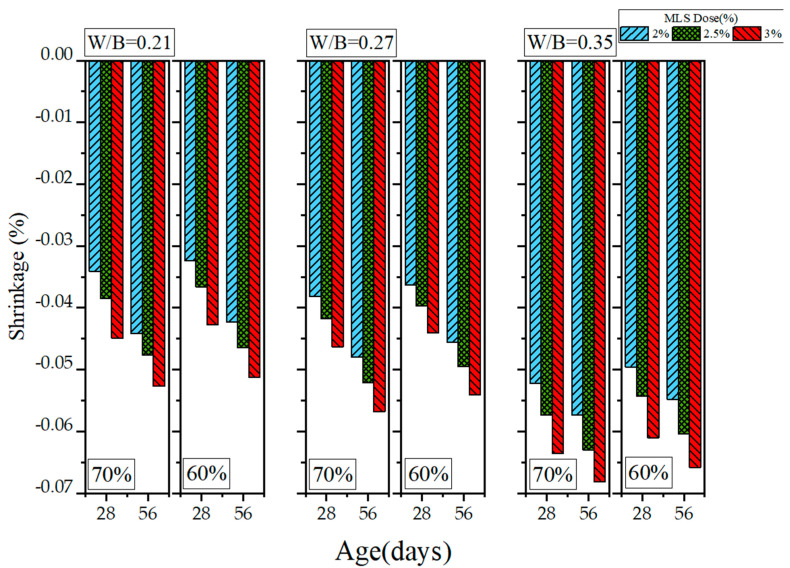
Length changes of green and ecofriendly grouting material with MLS.

Because HPC has a low W/B ratio, it exhibits drying shrinkage, a problem that many scholars have attempted to solve. In this study, length change experiments were conducted to examine the drying shrinkage of the GEGM. The results are presented in Figure 19, Figure 20 and Figure 21. As shown in Figure 19, when the W/B ratio was 0.21 and when 2% to 3% PCE was added, the length change was within the range of −0.0331% to −0.0371%. When the W/B ratio was increased from 0.21 to 0.27, the length change reached −0.034% to −0.0391%, an increase of approximately 2.71% to 7.9%. When the W/B ratio was further increased from 0.27 to 0.35, the length change reached −0.0481% to −0.0571%, an increase of approximately 29.8% to 49.8%. When the W/B ratio was low, the changes in drying shrinkage were small, presumably because the water used was able to completely hydrate the materials. Therefore, once the curing duration reached 56 days, the drying shrinkage was minimal. Regarding the sand content, MLS analysis revealed that adding 2% to 3% MLS, curing it for 28 days, and selecting s sand content of 70% all contributed to a length change of −0.0341% to −0.0449%. However, selecting a sand content of 60% (while holding the other variables constant) contributed to a length change of −0.0324% to −0.0427%, a decrease of 4.89% to 4.98%. Once the curing duration reached 56 days, selecting a sand content of 70% contributed to a length change of −0.0442% to −0.0489%. However, selecting a sand content of 60% (while keeping the other variables constant) contributed to a length change of −0.0423% to −0.0465%, a decrease of 4.29% to 4.9%. The drying shrinkage with 60% sand content was approximately 4.29% to 4.9% lower than that with 70% sand content, because the higher sand content increased the overall compactness of particle distribution and increased the slurry’s contraction.

#### 3.3.3. Four-Terminal Resistance of the GEGM

**Figure 22:**
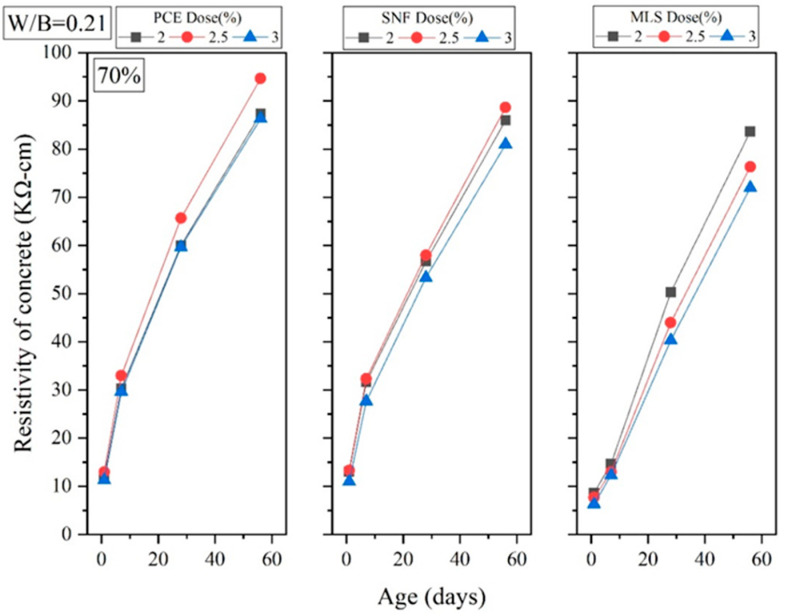
Four-terminal resistance of green and ecofriendly grouting material with sand content of 60%.

**Figure 23:**
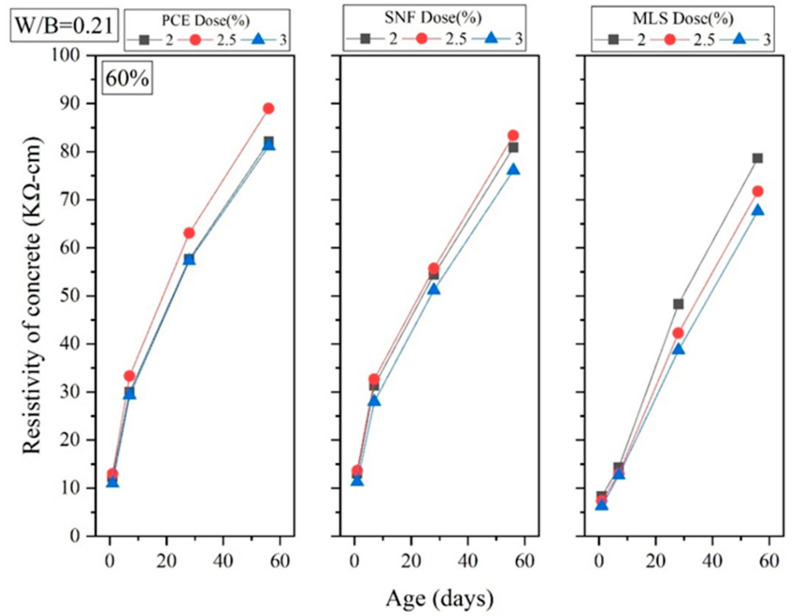
Four-terminal resistance of green and ecofriendly grouting material with sand content of 70%.

Four-terminal resistance measurement is a nondestructive test which uses resistivity to determine the number of pores in specimens and subsequently determine their durability. This test is based on the fact that a higher electrical resistance indicates a higher density, and density and durability are correlated. Experiments were conducted in this study to obtain the electrical resistance of GEGM specimens and assess the correlation between electrical resistance and water absorption rate. As shown in Figure 22 and Figure 23, the longer the curing duration, the greater the electrical resistance and the lower the water absorption rate. This means that the higher the electrical resistance, the greater the density. When curing was performed for 1–7 days, no correlation was observed between the sand content and electrical resistance. However, once the curing duration reached 28–56 days, the specimens became denser, and the electrical resistance, as a result of the sand content and porosity being different, increased. Sand content of 70% resulted in the highest density, thereby optimizing the packing performance. When the W/B ratio was 0.21, when 2% to 3% PCE was added, when curing was performed for 1 day, and when the sand content was 70%, the electrical resistance was 11.33 to 13 kΩ·cm. When the sand content was decreased from 70% to 60% (while keeping the other variables constant), the electrical resistance reached approximately 11 to 12.33 kΩ·cm, a minimal change. When curing was performed for 7 days, sand content of 70% resulted in an electrical resistance of approximately 29.67 to 33 kΩ·cm. When the sand content was decreased from 70% to 60%, the electrical resistance reached approximately 29.33 to 33.33 kΩ·cm, a minimal change. When curing was performed for 28 days, a sand content of 70% resulted in an electrical resistance of 59.67 to 65.66 kΩ·cm. When the sand content was decreased from 70% to 60%, the electrical resistance reached approximately 57.33 to 63.67 kΩ·cm, a decrease of 3% to 4%. When curing was performed for 56 days, a sand content of 70% resulted in an electrical resistance of 86.33 to 94.67 kΩ·cm. When the sand content was decreased from 70% to 60%, the electrical resistance reached 80.67 to 88.67 kΩ·cm, a decrease of 6.33% to 6.56%.

## 4. Conclusions

1. The optimal GEEPGM formula was obtained by adding 2.5% PCE at a W/B ratio of 0.21.
2. The use of a high level of sand content increased the durability of the GEEPGM but had minor effects on its workability and mechanical properties.
3. Compressive strength and the water absorption rate were used to build a regression model. The results indicated that *y* = 9.59 − 0.098*x* and *R*^2^ = 0.78, verifying the reliability of the results.
4. Compressive strength and the ultrasonic wave speed were used to build a regression model. The results indicated that *y* = −4.75 − 0.0051*x* + 3.46 × 10^−6^*x*^2^ and *R*^2^ = 0.88, verifying the reliability of the results.
5. The optimal amounts of PCE, SNF, and MLS to be added were 2.5%, 2.5%, and 2%, respectively.
6. The addition of an excessive amount of MLS considerably increased the setting time and affected the hydration results, which compromised the engineering properties of the GEEPGM.

## Figures and Tables

**Table 1:** Basic composition analysis of cementitious materials of green and ecofriendly grouting material (wt.%).

Chemical Composition (%)		Cement	FA	GGBFS
SiO_2_		21.31	50.5	34.42
Al_2_O_3_		4.58	24.7	14.35
Fe_2_O_3_		2.87	7.4	0.29
CaO		65.37	2.6	39.67
MgO		1.18	1.5	7.75
SO_3_		2.13	0.8	0.57
Na_2_O		0.26	0.8	0.24
K_2_O		0.62	3.0	0.28
**Physical property**	Fineness (m^2^/kg)	321	381.8	400
	Specific gravity	3.15	2.16	2.90
	LOI	2.65	1.70	-

**Table 2:** Mix proportions of green and ecofriendly grouting material.

W/B	Sand Content (%)	Coarse Aggregates (kg/m^3^)	Fine Aggregates (kg/m^3^)	SP		Water (kg/m^3^)	FA	GGBFS	Cement
				Adding (%)	(kg/m^3^)		(kg/m^3^)	(kg/m^3^)	(kg/m^3^)
0.21	70	451.77	1038.25	2	15.16	144.03	114.62	151.09	492.33
		451.77	1038.25	2.5	18.95	140.24			
		451.77	1038.25	3	22.74	136.45			
	60	602.36	889.93	2	15.16	144.03			
		602.36	889.93	2.5	18.95	140.24			
		602.36	889.93	3	22.74	136.45			
0.27	70	451.77	1038.25	2	13.61	170.15	102.91	135.65	442.05
		451.77	1038.25	2.5	17.02	166.75			
		451.77	1038.25	3	20.42	163.35			
	60	602.36	889.93	2	13.61	170.15			
		602.36	889.93	2.5	17.02	166.75			
		602.36	889.93	3	20.42	163.35			
0.35	70	451.77	1038.25	2	11.98	197.68	90.58	119.39	389.06
		451.77	1038.25	2.5	14.98	194.69			
		451.77	1038.25	3	17.97	191.69			
	60	602.36	889.93	2	11.98	197.68			
		602.36	889.93	2.5	14.98	194.69			
		602.36	889.93	3	17.97	191.69			

## Data Availability

The study did not report any data availability statement; all data were sourced from real experiments.
